# Integrated TCGA analysis implicates lncRNA CTB-193M12.5 as a prognostic factor in lung adenocarcinoma

**DOI:** 10.1186/s12935-018-0513-3

**Published:** 2018-02-22

**Authors:** Xuehai Wang, Gang Li, Qingsong Luo, Jiayong Xie, Chongzhi Gan

**Affiliations:** Department of Thoracic Surgery, Sichuan Academy of Medical Sciences and Sichuan Provincial People’s Hospital, 32 West Second Section First Ring Road, Chengdu, 610072 Sichuan People’s Republic of China

**Keywords:** Lung cancer, lncRNA, Prognostic factor, TCGA datasets

## Abstract

**Background:**

Lung cancer is a malignant tumor with the highest incidence and mortality around the world. Recent advances in RNA sequencing technology have enabled insights into long non-coding RNAs (lncRNAs), a previously largely overlooked species in dissecting lung cancer pathology.

**Methods:**

In this study, we used a comprehensive bioinformatics analysis strategy to identify lncRNAs closely associated with lung adenocarcinoma, using the RNA sequencing datasets collected from more than 500 lung adenocarcinoma patients and deposited at The Cancer Genome Atlas (TCGA) database.

**Results:**

Differential expression analysis highlighted lncRNAs CTD-2510F5.4 and CTB-193M12.5, both of which were significantly upregulated in cancerous specimens. Moreover, network analyses showed highly correlated expression levels of both lncRNAs with those of differentially expressed protein-coding genes, and suggested central regulatory roles of both lncRNAs in the gene co-expression network. Importantly, expression of CTB-193M12.5 showed strong negative correlation with patient survival.

**Conclusions:**

Our study mined existing TCGA datasets for novel factors associated with lung adenocarcinoma, and identified a largely unknown lncRNA as a potential prognostic factor. Further investigation is warranted to characterize the roles and significance of CTB-193M12.5 in lung adenocarcinoma biology.

**Electronic supplementary material:**

The online version of this article (10.1186/s12935-018-0513-3) contains supplementary material, which is available to authorized users.

## Background

Lung cancer is the leading cause of cancer-related mortality worldwide, with a particularly low 5-year survival rate for patients suffering from this disease at its advanced stages. In the US, lung cancer is estimated to account for approximately one quarter (26%) of all cancer-related deaths in the year 2017 [1]. In China, which currently hosts the largest population in the world, 730,000 new cases of lung cancer were estimated for the year 2015, along with more than 610,000 deaths [2]. Across the globe, as incidence and mortality generally continue with rise, lung cancer has become a major public health problem, and is therefore under intensive biomedical and clinical research.

Breakthroughs in ‘omics’ technologies, such as genomics, transcriptomics, and proteomics, have opened avenues for a systematic approach for understanding and treating cancer [3, 4]. In particular, a flurry of recent cancer profiling studies have focused on RNA sequencing (RNA-Seq), a rapidly maturing development of the next-generation sequencing technology. Compared with microarray analysis, RNA-Seq profiling allows for larger dynamic range, and higher sensitivity and throughput [5]. As a result, RNA-Seq profiling has been used in several recent studies of lung cancer molecular pathogenesis, including discovery of novel mutations in key oncogenes and genomic rearrangements in squamous cell lung cancer [6] and adenocarcinoma [7], identification of potential biomarkers in non-small cell lung cancer (NSCLC) [8], and quantification of expression of marker genes [9].

One revelation largely enabled by high-throughput sequencing analysis was that non-coding RNAs make up the majority (approx. 85%) of transcriptome. Based on transcript length, non-coding RNAs can be divided into short non-coding RNAs (sncRNAs, < 200 nucleotide) and long non-coding RNAs (lncRNAs, > 200 nucleotide) [10]. Deregulation of lncRNAs has been well recognized in cancer, and has been suggested to modulate tumor development at chromosomal, transcriptional, and post-transcriptional levels [10, 11]. In lung cancer, the list of implicated lncRNAs is expanding rapidly [11]. However, much still remains unknown about the mechanics and significance of lncRNAs in many aspects of this disease, such as carcinogenesis, development, metastasis, response to anti-cancer treatment, and prognosis.

In this study, we took advantage of large-scale expression profiles and a systems biology strategy to identify lncRNAs that were significantly regulated in lung cancer specimens, and were strongly co-expressed with a large pool of protein-coding genes (PCGs). In order to detect co-expression pattern among the lncRNAs and PCGs in our TCGA datasets, weighted gene co-expression network analysis (WGCNA) was applied. WGCNA has been established as an effective data mining method for finding clusters or modules of highly correlated biomolecules and identifying intramodular “hubs”, including genes [12], miRNAs [13], and metabolites [14]. Consequently, WGCNA has been successfully applied in several lung cancer profiling investigations, such as identification of differential mRNA expression [12] and lncRNAs expression profile signature [15] in lung squamous cell carcinoma.

In the present study, we used RNA-Seq datasets from The Cancer Genome Atlas (TCGA) database to identify novel lncRNAs associated with lung cancer. LncRNA profiling and protein-coding transcript profiles of lung cancer were extracted from TCGA. Afterwards, these datasets were subjected to a battery of analyses, including differential expression analysis, co-expression network and cluster analyses, KEGG pathway enrichment, and survival analysis. After several rounds of screening, two largely uncharacterized lncRNAs, CTD-2510F5.4 and CTB-193M12.5, were identified. Both lncRNAs were significantly upregulated in cancerous specimens and co-expressed with 304 protein-coding genes, suggesting a wide spectrum of target PCGs under the modulation of these two lncRNAs. More importantly, expression levels of CTB-193M12.5 also showed significant negative correlation with the prognosis of the patients from whom the RNA-seq datasets were derived. Together, our results provide a promising lncRNA candidate for further validation and characterization by “wet bench” and clinical research.

## Methods

### Data collection and preprocessing

The data used in this study were obtained from The Cancer Genome Atlas database (https://portal.gdc.cancer.gov/), including protein-coding transcript and lncRNA profiles of lung adenocarcinoma specimens and the corresponding patient clinical follow-up data. RNA-Seq data (presented as Fragments Per Kilobase Million) were collected on Illumina HiSeq platforms.

The two datasets came from a total of 592 specimens, which consisted of 59 normal and 533 cancerous tissues. Notably, there are 57 pairs of cancerous and the corresponding adjacent tissue in the datasets. Before further processing, quantile normalization was performed on the ‘Level-3’ read counts to standardize the data.

Next, we selected lncRNAs and PCGs whose normalized FPKM values were larger than 1 (in RNA-seq analyses, genes with a FPKM value no great than 1 are typically considered as not expressed) in more than 50% of all 57 specimen pairs, and extracted the expression of these retained lncRNAs and PCGs from all 593 specimens for further analysis.

### Screening for differentially expressed lncRNAs and protein-coding genes

Expression profiles of lncRNAs and PCGs were analyzed separately, in order to identify the differential expression of these genes in normal and cancerous tissue samples. A previously reported approach was used in screening for differentially expressed genes [16]. Briefly, for lncRNAs and PCGs with an expression of 0 in more than 30% of either normal or cancerous tissues, Filter B was applied, while Filter A was applied for the remaining lncRNAs and PCGs.

Filter A. fold_change > 2 or fold_change < 0.5 and statistically significant (p < 0.01, paired Student’s t test), where fold_change values calculated as indicated in Table 1. A fold_change value of greater than two indicates that compared with normal specimen, expression of the gene is upregulated in the cancerous specimens, whereas a fold_change of less than 0.5 indicates downregulated expression in cancerous specimens.

**Table 1:** Calculation of fold_change values for lncRNAs and PCGs screened with Filter A

	Cancerous sample	Normal sample
Number of samples where expression is 0	A1	B1
Number of samples where expression is not 0	A2	B2

fold_change = (A2 * (B1 + B2)/B2 * (A1 + A2)) > 2

Filter B. fold_change > 2 or fold_change < 0.5 and statistically significant (p < 0.01, Fisher’s exact test), where fold_change values were calculated as fold_change = non-zero expression in cancerous specimen/non-zero expression in normal specimen.

Subsequently, we performed hierarchical cluster analysis using the R package heatmaps. Based on the z-scores derived for the expression levels of selected genes in all samples, we calculated the Euclidean distances of all gene pairs, which were then used to detect gene clusters.

### Co-expression network analysis of the expression of lncRNAs and PCGs

For detection of gene co-expression modules, co-expression network analysis was performed on both expression profiles using an R package WGCNA [17].

Briefly, following FPKM normalization, the Pearson’s correlation coefficient (PCC) *cor*(*i*, *j*) was calculated for each pair of retained lncRNAs and PCGs from the corresponding expression levels. Next, a similarity co-expression matrix was computed as follows:

$$ a_{ij} = \left( {0.5 \times \left( {1 + cor\left( {i,j} \right)} \right)} \right)^{\beta } $$

where $a_{ij}$ represents connection strength between nodes i and j.

Afterwards, the similarity matrix was transformed to an adjacency matrix (AM) using a power β = 14, based on the scale-free topology criterion described in the WGCNA package documents [17]. Then, a topological overlap matrix (TOM) was derived from the AM, and was in turn converted into a dissimilarity TOM, from which a dendrogram was mapped via hierarchical clustering. By applying the dynamic tree cutting technique, clusters were obtained from the dendrogram. The resulting clusters are co-expression modules containing lncRNAs and PCGs that are considerably interconnected.

### Analysis of correlation between co-expression modules and clinical status

After identifying co-expression modules, we selected the Blue module, a co-expression of five lncRNAs and 304 PCGs, as our evidence suggested that it was the module most positively correlated with lung cancer. Then, through clustering of PCCs, two lncRNAs closely correlated to PCGs were selected from the Blue module. In parallel, we also screened for PCGs in the module based on the strength of their correlation with the five lncRNAs. The PCGs were classified into two groups, namely those with high level of correlation to the five lncRNAs, and those with low level of correlation. The former group was then selected for KEGG pathway enrichment analysis.

### Kaplan–Meier survival analysis

To investigate the impact of the expression levels of two candidate lncRNAs on prognostic survival of patients, Kaplan–Meier survival analysis was performed using GEPIA (http://gepia.cancer-pku.cn/), a web-based interactive toolkit for analyzing gene expression profiling datasets [18]. We compared the prognostic survival of patients groups based on the expression level of the either lncRNA. Briefly, patients were assigned into either the high expression or low expression group based on the expression level of each lncRNA in their specimens, and the prognostic survival was analyzed using the survival analysis feature with default parameters.

To further validate the results, another web-based interactive toolkit, Kaplan–Meier plotter was applied [19]. Kaplan–Meier plotter (http://kmplot.com/analysis/) is a comprehensive online platform that offers assessment of the effect of 54,675 genes on survival based on 10,293 cancer samples. In particular, we focused on the dataset of 2437 lung cancer patients with a mean follow-up of 49 months. We selected all databases related to NSCLC (GSE1918, GSE29013, GSE30219, GSE31210, GSE3141, GSE37745, GSE50081), which included a total of 673 patients, to assess the prognostic value of the two candidate lncRNAs in lung adenocarcinoma carcinoma.

### LncRNA function predication and target gene enrichment analysis

Next, we used RIblast, an RNA–RNA interaction prediction algorithm package to predict target mRNAs [20]. Using a seed-and-extension approach, RIblast discovers seed regions using suffix arrays, and extends these regions based on an RNA secondary structure energy model. We used 27,519 mRNA sequences obtained from The RefGene database (http://varianttools.sourceforge.net/Annotation/RefGene) to establish the RIblast dataset. The predicted target genes were sorted by sum_energy, and the top 100 genes were selected for GO enrichment analysis.

## Results

### Preprocessing of the datasets

We used RNA-seq datasets (presented as FPKMs) collected from 592 specimens consisting of 533 cancerous and 59 normal specimens, including 57 pairs of matched cancerous and normal adjacent tissue samples. Moreover, the datasets contain expression levels of 14,448 lncRNAs and 19,069 protein-coding genes. Upon obtaining the expression profiles, we performed quantile normalization to standardize the datasets. Afterwards, we selected lncRNAs and PCGs whose expression levels are greater than 1 in more than 50% of the 57 matched specimen pairs. After the selection, a total of 679 lncRNAs and 12,040 PCGs were used for further analysis.

### Expression analysis of lncRNAs and PCG

**Fig. 1:**
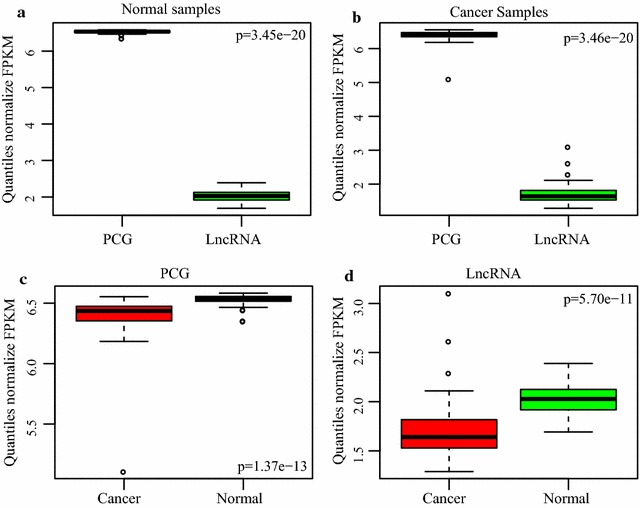
Comparison between global expression levels of PCGs and lncRNAs in normal and lung cancer biopsies. PEG and lncRNA expression levels are shown in **a** normal and **b** cancerous samples. Expression levels of **c** PCGs and **d** lncRNAs between normal and cancerous samples were also compared

We compared the expression levels of lncRNA against those of PCGs in both normal (Fig. 1a) and in cancerous tissue samples (Fig. 1b). In both types of tissues, expression levels of lncRNAs are much lower than those of PCGs, which is consistent with previous reports [21, 22]. Furthermore, we compared global expression differences in the expression of PCGs (Fig. 1c) and lncRNAs (Fig. 1d) in both types of specimens. As shown in these figures, both PCG and lncRNA showed significant differences. In particular, the expression of PCGs and lncRNAs in cancerous specimens are generally low. In terms of expression levels between normal and cancerous specimens, lncRNAs showed grater variance than PCGs, suggesting an interesting possibility that lncRNA expression is more specific, whereas PCGs are expressed more stably, between normal and cancerous states in lung cancer. Moreover, this higher level of specificity lends lncRNAs to be more suitable targets for targeted therapy of lung adenocarcinoma.

### Analysis of differential expressions of PCG and lncRNAs

As described in a previous section, 679 lncRNAs and 12,040 PCGs were retained for differential expression analysis, during which fold change of the expression level of each PCG or lncRNA was calculated as aforementioned. A total of 119 differentially expressed lncRNAs and 1934 PCGs were identified. Table 2 presents an overview of the numbers of differentially expressed lncRNAs and PCGs. Interestingly, while comparable numbers of differentially expressed PCGs showed significant up- or down-regulation, the majority of differentially expressed lncRNAs was downregulated in cancerous specimens as compared with normal ones, suggesting that in lung cancer, lncRNAs are more inclined to be downregulated.

**Table 2:** An overview of differentially expressed lncRNAs and PCGs

	lncRNAs	PCGs
Upregulated in lung cancer	29	899
Downregulated in lung cancer	90	1035
Total	119	1934

**Fig. 2:**
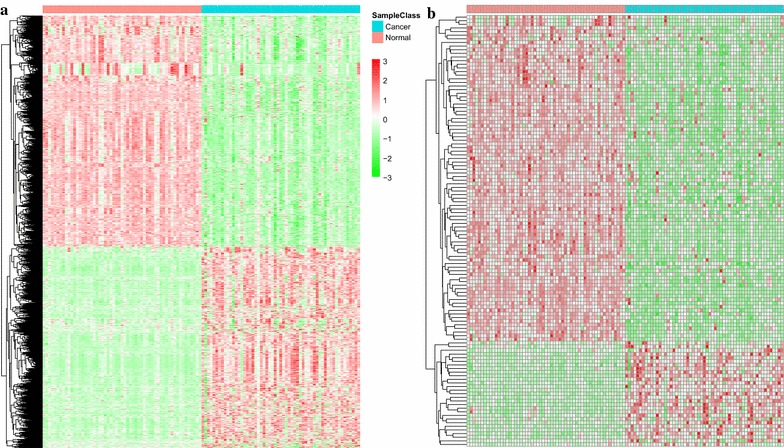
Heatmaps showing clustering patterns of differentially expressed **a** PCGs and **b** lncRNAs between normal and lung cancer specimens

Next, we performed clustering analysis on these differentially expressed lncRNAs and PCGs. As shown in the resulting heatmaps, differentially expressed lncRNAs (Fig. 2a) and PCGs (Fig. 2b) consistently distinguished normal specimens from cancerous ones.

### KEGG enrichment analysis

**Fig. 3:**
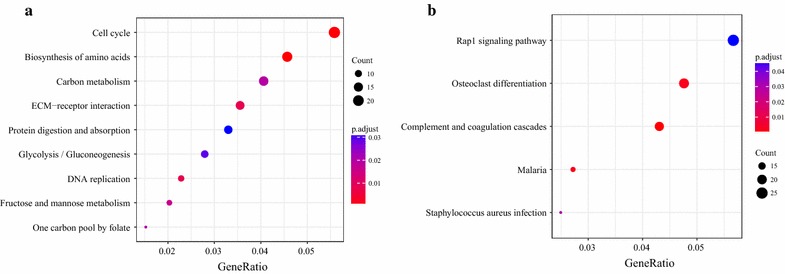
KEGG enrichment analysis of protein-encoding genes that are significantly **a** upregulated and **b** downregulated in lung cancer samples as compared with normal samples

After identifying differentially expressed PCGs, we performed a KEGG pathway enrichment analysis using the R package clusterProfiler for overview of the biological significance of these genes [23]. As shown in Fig. 3, nine KEGG pathways were enriched among significantly upregulated genes, which encompass a variety of cellular processes, including cell cycle, DNA replication, ECM-receptor interaction, and several metabolism-related pathways. On the other hand, five KEGG pathways were enriched among downregulated genes, including signaling cascades such as Rap1 signaling pathway and the complement and coagulation cascades.

### Identification of co-expression modules

**Fig. 4:**
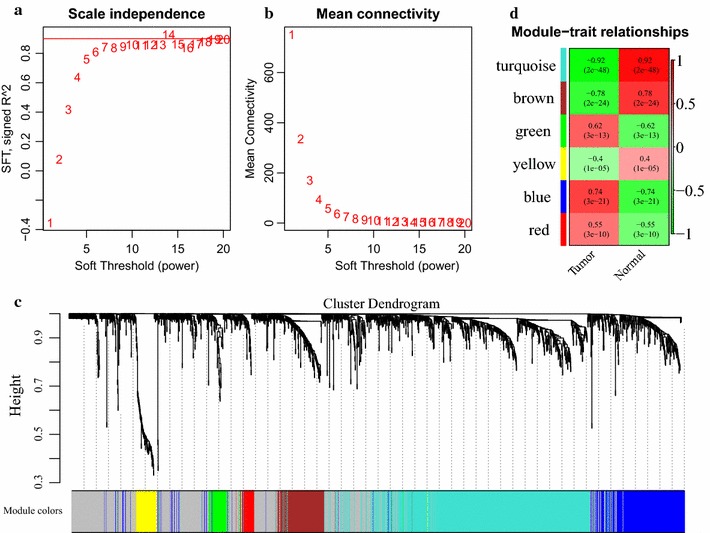
An overview of co-expression modules identified from the lung cancer RNA-seq datasets used in this study. **a** Scale independence and **b** mean network connectivity for different soft-thresholding powers (β). A soft-thresholding power of 14 was selected to achieve maximal model fit. **c** Cluster dendrogram of the identified lncRNA-PEG co-expression modules. Each of the differential expressed lncRNAs and PCGs is represented by a tree leaf, and each of the six modules by a tree branch. The lower panel shows colors designated for each module. Note that the leaves presented in the color gray indicates unassigned lncRNAs and PCGs. **d** Module-trait weighted correlations and corresponding p values (in parenthesis) between the identified modules and clinical status (normal and tumor). The color scale on the right shows module-trait correlation from − 1 (green) to 1 (red), where green represents a perfect negative correlation, and red a perfect positive one

lncRNAs have been known to regulate gene expression in a number of ways, assuming roles including decoys, scaffolds, guides, and signals [10]. We postulate that for an lncRNA to regulate the expression of a PCG, their expression profiles are expected to exhibit similar patterns. Therefore, using the R package WGCNA, we mapped a weighted co-expression network of lncRNAs and PCGs and identified co-expression modules. Although the WGCNA approach has been highly automated through continued algorithm optimization, several key parameters still needed to be fine-tuned empirically in order to ensure that the co-expression network to be constructed is scale-free [17]. To this end, we finally determined a β value of 14 (Fig. 4a, b).

Subsequently, the expression matrix was transformed a topological overlap matrix, to which we applied the average-linkage method for sequence clustering. Next, we employed a dynamic tree cutting procedure to detect co-expression clusters (i.e. modules). Again, after optimization, the minimal number of genes in each cluster was set at 30 in order to fulfill the criteria of dynamic tree cutting. Afterwards, another round of clustering analysis (height = 0.25) was performed, where closely associated modules were merged into larger ones.

In the end, WGCNA analysis identified six co-expression modules (Fig. 4c). Table 3 summarizes the distribution of lncRNAs and PCGs among these modules. Altogether, a co-expression network of 1303 PCGs and 89 lncRNAs was constructed. Notably, we also computed and plotted the correlation of each module with the clinical status of the corresponding samples, as a measure the strength of correlation between the lncRNAs and PCGs in that module and lung cancer. As shown in Fig. 4d, the Blue module showed the strongest positive correlation (module-trait weighted correlation = 0.74) with cancerous specimens, and the turquoise module a negative correlation that is close to perfect (module-trait weighted correlation = − 0.92).

**Table 3:** A summary of the six co-expression modules revealed with WGCNA analysis

Module color	No. lncRNAs	No. PCGs
Blue	5	304
Brown	13	141
Green	1	63
Red	1	39
Yellow	4	67
Turquoise	45	709

**Fig. 5:**
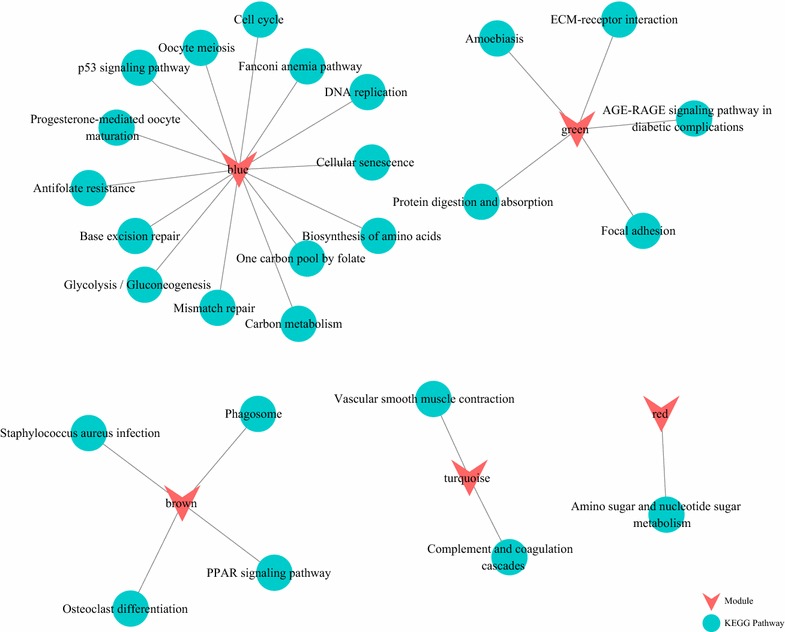
KEGG pathway analysis of the six modules

Next, we performed a second KEGG enrichment analysis for each co-expression module with clusterProfiler. From the 1303 PCGs in the six modules, a total of 26 KEGG pathways were enriched. As shown in Fig. 5, a distinct set of pathways were enriched from each module, with no overlapping, suggesting largely independent sets of functions exerted by genes in each co-expression module. Furthermore, by comparing with the KEGG pathways enriched from differentially expressed genes, we noticed that six out of the nine pathways enriched in upregulated genes were also enriched from the Blue module. As described above, our correlation analysis indicates that this co-expression module shows the strongest positive correlation with cancerous specimens. Moreover, among the 14 KEGG pathways enriched from this module, many have been established as key cascades closely related to the initiation, growth, and dissemination of lung cancer, including cell cycle and senescence, DNA damage repair, and p53 signaling [24–26]. These enriched KEGG pathways suggest high relevance of the genes in the Blue module to lung cancer.

### Analysis of the correlation between lncRNAs and PCGs in the Blue module

**Fig. 6:**
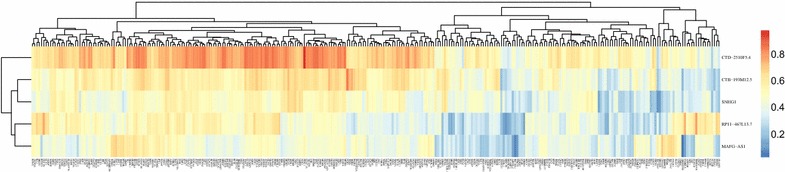
Pearson’s correlation coefficients (PCCs) of expression levels of each lncRNA-PEG pair within the Blue module were computed and clustered. In the resulting heatmap, the PCCs of each of the five lncRNAs with the 304 PCGs were plotted in a separate row. The color scale on the right shows PCC value from 0 (blue) to 1 (red), where blue represents no correlation, and red a perfect positive one. Euclidean distances were used for the clustering analysis

Due to its strong positive correlation with cancerous specimens, as well as the versatile enriched KEGG pathways of significance in lung cancer, we looked further into the Blue module. For all five lncRNAs and the 304 PCGs in the module, we extracted the expression level PCC for each lncRNA-PCG pair, and performed cluster analysis based on these PCCs. As shown in Fig. 6, two lncRNAs, namely CTD-2510F5.4 and CTB-193M12.5, showed the strongest overall co-expression with the PCGs, suggesting central roles for these lncRNAs in this co-expression module.

Next, we examined the annotated functions of 304 PCGs in the module. Based on overall strength with the five lncRNAs, we selected 178 PCGs (PCC > 0.6) and performed KEGG pathway enrichment analysis (Table 4). Notably, out of the 15 KEGG pathways enriched, a predominant majority (13 pathways) overlapped with those enriched from all PCGs in the module, suggesting that these 178 PCG are representative of the major functions of the protein-coding genes in the Blue module.

**Table 4:** KEGG pathway enrichment analysis of PCGs with a high overall correlation with the five lncRNAs in the Blue co-expression module

Description	GeneRatio	p value	q value	Count
Cell cycle	15/79	2.71E−12	2.56E−10	15
DNA replication	8/79	3.18E−09	0.000000151	8
Biosynthesis of amino acids	9/79	7.29E−08	0.0000023	9
Carbon metabolism	10/79	0.000000378	0.00000895	10
Glycolysis/Gluconeogenesis	8/79	0.000000524	0.00000992	8
One carbon pool by folate	5/79	0.00000181	0.0000286	5
Fanconi anemia pathway	5/79	0.000302021	0.003766033	5
Cellular senescence	8/79	0.000318021	0.003766033	8
Oocyte meiosis	7/79	0.000369272	0.003887073	7
Progesterone-mediated oocyte maturation	6/79	0.000679229	0.0064348	6
p53 signaling pathway	5/79	0.000810879	0.006983646	5
Mismatch repair	3/79	0.00186508	0.014724313	3
Pentose phosphate pathway	3/79	0.004048799	0.029505417	3
Antifolate resistance	3/79	0.004447883	0.030098454	3
Fructose and mannose metabolism	3/79	0.005315566	0.033571995	3

### Analysis of the expression level of lncRNAs in the Blue module

**Fig. 7:**
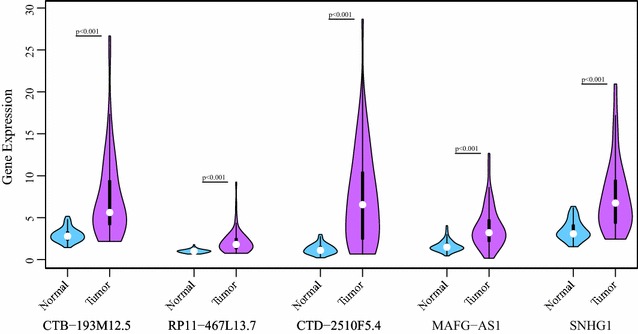
All five lncRNAs in the Blue module showed significant upregulation in lung cancer samples (p < 0.001 for all, Mann–Whitney test)

We analyzed the expression levels of the five lncRNAs in the Blue module between cancerous and normal tissues (Fig. 7). All five lncRNAs were significantly upregulated in lung cancer samples as compared with normal samples (p < 0.001 for all, Mann–Whitney test). In particular, CTD-2510F5.4 and CTB-193M12.5 showed most intense upregulation.

### Construction of an lncRNA-PCG regulatory network

**Fig. 8:**
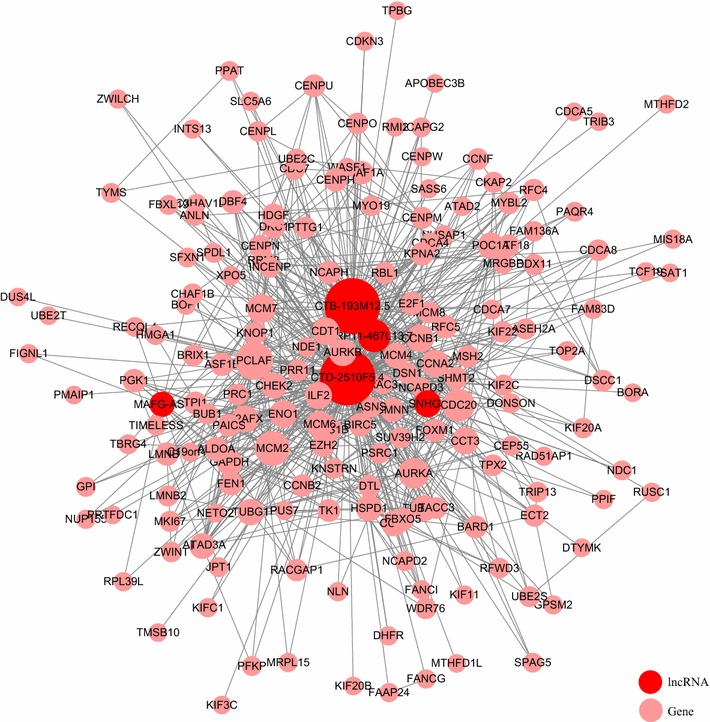
An interaction network constructed with the five lncRNAs and 178 highly correlated PCGs (Pearson’s correlation coefficient > 0.6) in the Blue module

Next, we set out to construct an lncRNA-PCG regulatory network of the five lncRNAs in the Blue module and the 178 highly correlated PCGs selected in the previous section. Protein–protein interaction data were retrieved from Human Integrated Protein–Protein Interaction rEference database and visualized with Cytoscape. A regulatory network with 683 connections and 182 nodes was constructed. As shown in Fig. 8, the majority of the connections concentrated on a few nodes, suggesting significant roles of the corresponding lncRNAs and PCGs. Notably, two lncRNAs, CTD-2510F5.4 and CTB-193M12.5, had 172 and 81 connections, respectively (Table 5). These connections constituted approx. 25 and 12% of all connections in the regulatory network, which pointed to the centrality of these two ‘hub’ lncRNAs.

**Table 5:** Nodes with more than 15 connections in the network shown in Fig. 8

Node	Degree	Type
CTD-2510F5.4	172	lncRNA
CTB-193M12.5	81	LncRNA
PCLAF	33	PCG
MCM2	28	PCG
CDC20	24	PCG
RP11-467L13.7	24	LncRNA
AURKA	21	PCG
MCM7	18	PCG
AURKB	16	PCG
CCNA2	16	PCG
CCNB1	16	PCG
CDT1	16	PCG
ENO1	16	PCG

**Fig. 9:**
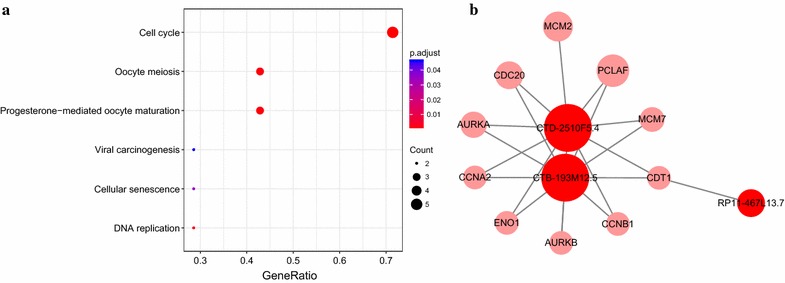
**a** KEGG pathways enriched from the ten PCGs selected from the lncRNA-PEG interaction network. **b** An interaction network between these ten PCGs and all five lncRNAs in the Blue module

To highlight the highly connected genes, we selected only nodes with more than 15 connections. These nodes corresponded to three lncRNAs and ten PCGs (Table 5). From the ten PCGs, six KEGG pathways were enriched (Fig. 9a), which encompass essential aspects of cancer biology, including cell cycle and senescence, DNA replication, and viral carcinogenesis [24, 27, 28].

A closer analysis of the correlation between the lncRNAs and ten PCGs revealed that all ten PCGs were significantly correlated to CTD-2510F5.4, nine to CTB-193M12.5, and one to RP11-467L13.7 (Fig. 9b). The strong correlation of CTD-2510F5.4 and CTB-193M12.5 to these genes suggested strongly roles of these two lncRNAs in regulating the expression of these PCGs, which in turn, modulate the cancer initiation and development through a host of cellular processes, such as cell cycle and death, and DNA replication.

### Prognostic analysis of CTD-2510F5.4 and CTB-193M12.5 expression levels and patient survival

**Fig. 10:**
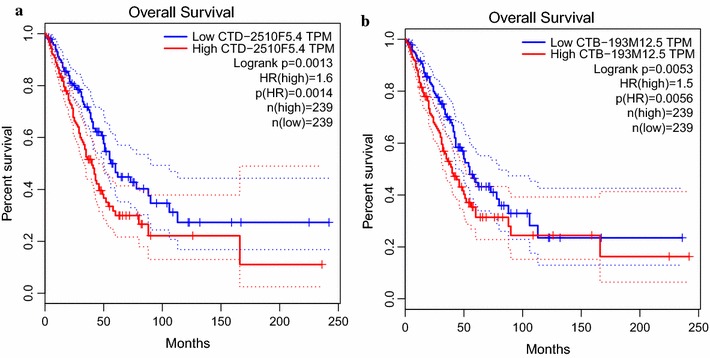
Kaplan–Meier analysis of lung adenocarcinoma-specific overall survival of patient (TCGA Datasets) with tumors expressing different levels of **a** CTD-2510F5.4 and **b** CTB-193M12.5. Dotted line indicates 95% confidence interval

Evidence so far suggest high relevance of CTD-2510F5.4 and CTB-193M12.5 in lung cancer, including dysregulated expression in and close correlation with the disease. To assess the clinical relevance of these lncRNAs, we performed prognostic survival analysis to examine whether the expression levels of these lncRNAs significantly correlate to the survival of patients who provided the specimens. As shown in Fig. 10, high expression of both lncRNAs were significantly negatively correlated with patient overall survival (OS; Logrank p = 0.0013 for CTD-2510F5.4; Logrank p = 0.0053 for CTB-193M12.5), suggesting potentials of both lncRNAs as prognostic indicators.

**Fig. 11:**
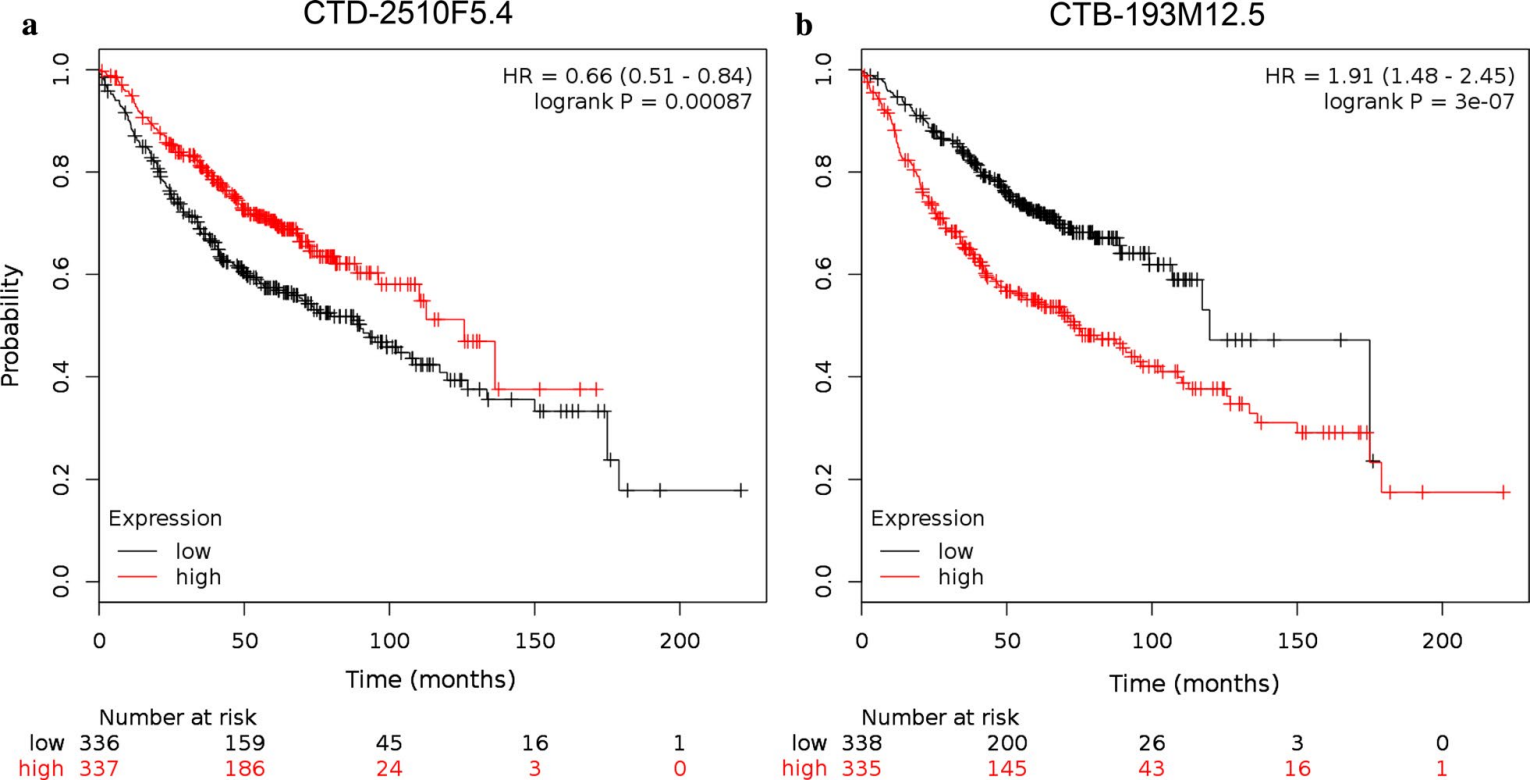
Kaplan–Meier analysis of lung adenocarcinoma-specific overall survival of 673 patients with tumors expressing different levels of **a** CTD-2510F5.4 and **b** CTB-193M12.5

To further validate the prognostic value of CTD-2510F5.4 and CTB-193M12.5 in lung cancer, an independent dataset consisting of 673 lung adenocarcinoma patients from seven GEO datasets was subjected to Kaplan–Meier survival analysis. As shown in Fig. 11, the two lncRNAs showed opposite direction of correlation of OS. Of note, in this analysis, CTD-2510F5.4 expression showed positive correlation with prognosis (Logrank p = 0.00087), which was inconsistent with our results. However, CTB-193M12.5 expression level was negatively correlated to patient OS (Logrank p = 3e−07), which was in accordance with our analysis of the TCGA profiles.

### CTB-193M12.5 target prediction and function analysis

**Fig. 12:**
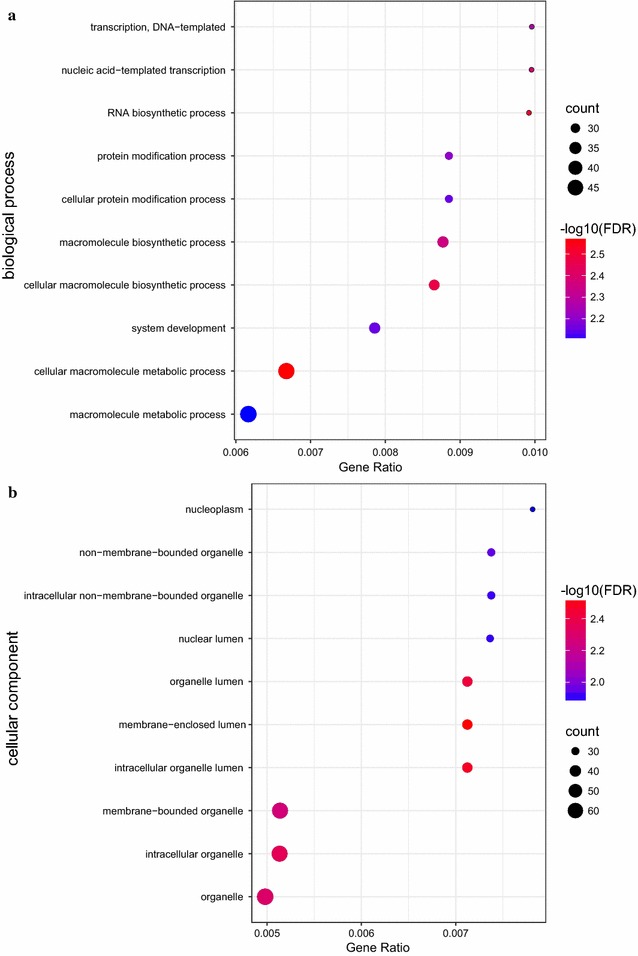
Dot plot of ten most significant pathway enriched from the predicted targets of CTB-193M12.5

Target genes of CTB-193M12.5 were predicted with RIblast, an RNA–RNA interaction prediction algorithm [20]. Based on the levels of intramolecular and intermolecular free energy between lncRNA-mRNA sequence, a list of target PCGs were generated (Additional file 1: Table S1). After sorting by sum_energy, the top 100 genes were subjected to GO enrichment analysis. No molecular function pathway was significantly enriched. Biological process pathway and cellular component pathway terms (false discovery rate < 0.05, gene number > 20) were sorted by significance, and the top ten enriched terms were retained (Fig. 12).

The most significantly enriched term was ‘cellular macromolecule metabolic process’, also with the greatest gene number. This term refers to chemical reactions and pathways involving macromolecules, including essential metabolic processes of DNA and glycoprotein. It is known that an important hallmark of cancer cells is a profound change in metabolism. Most tumor cells are characterized by higher rates of glycolysis, lactate production, and biosynthesis of lipids and other macromolecules [29]. These results hint at possible roles of CTB-193M12.5 in regulating lncRNAs implicated in DNA and/or glycoprotein metabolism.

## Discussion

Recent investigations have provided good evidence that opens avenues to the largely unknown roles of lncRNAs, which are estimated to make up for approximately 85% of the genome. More than 3000 lncRNAs have been identified so far; however, functions and biological roles for only 1% of them have been proposed, much fewer characterized [10]. Insights into the function of the few characterized lncRNAs suggest a surprising diverse variety of cellular processes, from chromatin modification, transcription, splicing, and translation to cellular differentiation, cell cycle regulation, and stem cells reprogramming [10].

Recent emergence and maturation of the RNA sequencing technology has greatly facilitated identifying lncRNAs associated with various diseases. Traditional hybridization-based approaches such as DNA microarray suffer from several limitations, including reliance on sequenced genomes, high background levels, and a relatively narrow dynamic range. More importantly, comparison of expression profiles across different experiments is often difficult and requires complex data processing. In contrast, RNA-Seq enjoys a number of advantages, including very low background signal and large dynamic range of detection. Furthermore, RNA-seq enables high-throughput sequencing of transcriptomes at single-base resolution, whose quantification across experiments can also be performed with simple normalization algorithms. Together, these factors have made RNA-seq an ideal choice for screening for lncRNAs with clinical significance.

Consequently, databases of publicly available RNA-seq profiles have been constructed and showing continuous growth, although many of the datasets remain to be mined with comprehensive bioinformatics tools in order to reveal identifies of potential key master regulators that could provide hints for validation and clinical application. In this study, we used transcriptome datasets collected with RNA-seq to screen for potential lncRNAs markers associated with lung cancer. The expression profiles were analyzed with a series of analytical tools. As a first step, lncRNAs and protein-coding genes that showed significant up- or down-regulation were identified (Fig. 1). From 592 specimens (59 normal and 533 cancerous specimens), 679 lncRNAs and 12,040 PCGs were selected for differential expression analysis, and 119 lncRNAs and 1934 PCGs were found to be differentially expressed. The large number of differentially expressed lncRNAs is consistent with the versatile roles and regulatory mechanisms of lncRNAs unveiled thus far, and suggests a vast unchartered territory of the roles of these biomolecules in lung cancer biology [10, 11, 30].

The next step was to detect similar patterns of expression among these differentially expressed lncRNAs and PCGs. There were two purposes to this analysis, namely to identify lncRNAs and PCGs that may function in pathways in the same cellular processes, and to identify lncRNAs (hubs) that potentially play central roles in modulating the expression of targets within the co-expression module [17, 31].

Unlike sncRNAs, lncRNAs are poorly conservative and highly versatile in modulating biomolecules. A plethora of mechanisms by which lncRNAs regulate gene expression have been reported [10]. Due to their large size and therefore the ability to adopt complex conformations, lncRNAs can bind to DNAs, RNAs, and proteins. These interactions, in turn, enable lncRNAs to act as activators, blockers, and scaffolds of their interacting partners, including DNA, mRNAs, miRNAs, transcription factors, and chromatin regulators [11]. At the transcriptional level, transcription of lncRNA upstream of a target can facilitate or impede that of the latter through modulating DNA conformation, RNA Pol III activity, or the association of transcription factors and promoters. In addition, lncRNAs also regulate alternative splicing, or serve as mRNA stabilizers and a sncRNA repertoire. Furthermore, lncRNAs can modulate genome activity through affecting histone modification, DNA methylation, and chromatin structure [10, 11, 32].

Of the 119 lncRNAs and 1934 PCGs that showed differential expression between normal and cancerous specimens, six co-expression modules were detected with weighted co-expression network analysis. Among these modules, the Blue module showed the strongest positive correlation with lung cancer (Fig. 4d). The five lncRNAs in this module, despite brief mentioning as part of significantly regulated genes in a handful of previous reports [33–38], remain almost entirely uncharacterized. Interestingly, all five lncRNAs showed upregulation in lung cancer specimens, suggesting potential tumor-promoting roles.

Similar to protein-coding genes, lncRNAs can be classified into two major groups, tumor suppressor lncRNAs and onco-lncRNAs [39]. Several lncRNAs have been proposed as oncogenic in lung cancer, including MALAT1 (a diagnostic and prognostic biomarker in NSCLC) [40], AK126698 (mediates cisplatin resistance in NSCLC) [41], and lncRNA-DQ786227 (implicated in chemical carcinogenesis) [42]. All three onco-lncRNAs showed upregulation in lung cancer, similar to the five lncRNAs in the Blue module. Conceivably, these lncRNAs may be novel onco-lncRNAs of clinical relevance to lung cancer, although further research is warranted for validation.

As for the 304 PCGs, KEGG pathway analysis showed that they were enriched in processes closely related to lung cancer biology, such as p53 signaling, cellular senescence, DNA replication, and metabolism [24, 25]. These enriched pathways may be used as a basis for gaining deeper insights into the five lncRNAs.

Following detection of co-expression networks, we chose the Blue module due to its strong correlation with lung cancer, and determined the hub genes in this module. To suppress background noise, 178 PCGs with strong over correlation with the five lncRNAs (PCC > 0.6) were selected and subjected to regulatory network analysis. Two lncRNAs, namely CTD-2510F5.4 and CTB-193M12.5, were identified as hubs of the resulting network. In addition, both lncRNAs also showed the strongest overall correlation with all 304 PCGs in the Blue module, further supporting their centrality in this co-expression module. Moreover, survival analysis showed significant correlation between expression of either lncRNA and poor prognostic overall survival, suggesting CTD-2510F5.4 and CTB-193M12.5 as potential prognostic indicators.

Currently very little is known about either lncRNA. As a result, neither has an official Human Genome Nomenclature Committee symbol. CTD-2510F5.4 (GenBank accession AC099850.7) is the transcript of the gene ENSG00000265415, which is located to chromosome 17 (chromosome 17: 59,065,973–59,264,225). CTD-2510F5.4 has been reported to show consistent increased expression in relation to p53 mutations in lung adenocarcinomas [33]. Moreover, CTD-2510F5.4 was also found to be differentially expressed in another study that used RNA-seq data from TCGA and two independent experiments of more than 60 lung adenocarcinoma specimens, which supports the validity of our results.

Functions of CTD-2510F5.4 remain to be characterized. Proline rich 11, a gene neighboring ENSG00000265415, was recently suggested as a weak prognostic factor in non-mucinous invasive lung adenocarcinoma [43], suggesting a possible mechanism by which elevated CTD-2510F5.4 expression contributes to poor prognosis. As suggested by KEGG pathway analysis, CTD-2510F5.4 may also be implicated in key lung cancer-related cellular processes such as senescence. Upon induction of cellular senescence with overexpression of oncogene B-RAF, CTD-2510F5.4 was shown to be downregulated as compared with control cells [34]. Since oncogene-induced senescence (OIS) is an important defense mechanism against lung cancer initiation [44], a hypothesis could be proposed, in which aberrant overexpression of CTD-2510F5.4 contributes to survival of cells overexpressing the tumor-promoting B-RAF despite OIS, and thereby exert oncogenic functions. More research is, obviously, needed for validation of this hypothetical mechanism.

The other hub lncRNA, CTB-193M12.5 (GenBank accession AC026401.7), is the product of the gene ENSG00000280206, which is located to chromosome 16 (chromosome 16: 15,570,622–15,708,653). CTB-193M12.5 was found to be upregulated in lung squamous cell carcinomas in a recent report analyzing RNA-seq profiles [37], which is consistent with our finding of the overexpression of this lncRNA in lung cancer specimens. In addition, expression of this lncRNA was reported to be dramatically increased in gastric cancer tissues [37] and in triple negative breast cancer cell lines and primary tumors (Cancer RNA-seq Nexus database, analysis title GSE58135) [45]. We also tried to gain insights into the potential functions of CTB-193M12.5 by predicting its target PCGs and enriched pathways. The most significantly enriched term suggests the roles of CTB-193M12.5 in DNA and/or glycoprotein metabolism, both are known to be crucial in cancer progression [29].

In summary, starting from TCGA gene transcript profiles collected from 592 lung cancer specimens, through integrated bioinformatics analyses, we identified two largely unknown lncRNAs CTD-2510F5.4 and CTB-193M12.5. Expression levels of both lncRNAs were significantly increased in lung cancer specimens, and showed strong correlation with those of more than 300 differentially expressed protein-coding genes. Moreover, further analysis placed these lncRNAs in the center of the regulatory network consisting of the lncRNAs and PCGs in a co-expression module that showed the strongest positive correlation with lung cancer. Most importantly, high expression of CTD-2510F5.4 and CTB-193M12.5 significantly correlated to poor overall prognostic patient survival, and the prognostic value of the latter was further supported by an independent validation.

Altogether, these results provide evidence that, for the first time, correlate CTB-193M12.5 with prognosis of lung cancer patients, and thereby can be used as the basis for further investigation towards elucidating its biological significance and clinical applications.

## Conclusions

Through mining existing TCGA datasets for novel factors, this study identified and validated a largely unknown lncRNA CTB-193M12.5 as a promising prognostic factor for lung adenocarcinoma.

## Additional file

**Additional file 1: Table S1.** CTB-193M12.5 target gene predicted by RIblast.

## Abbreviations

TCGA: The Cancer Genome Atlas
lncRNA: long non-coding RNA
RNA-Seq: RNA sequencing
NGS: next-generation sequencing
NSCLC: non-small cell lung cancer
PCG: protein-coding genes
WGCNA: weighted gene co-expression network analysis
FPKM: Fragments Per Kilobase Million
PCC: Pearson’s correlation coefficient
KEGG: Kyoto Encyclopedia of Genes and Genomes
